# Supplementary material for: E4F1 coordinates pyruvate metabolism and the activity of the elongator complex to ensure translation fidelity during brain development
Source: Nat Commun. 2025 Jan 2;16:67. doi: 10.1038/s41467-024-55444-y (PMC11696611; doi:10.1038/s41467-024-55444-y)
Supplement: Supplementary file 1 — Supplementary Information [file 41467_2024_55444_MOESM1_ESM.pdf]

Supplemental Figure S1

A

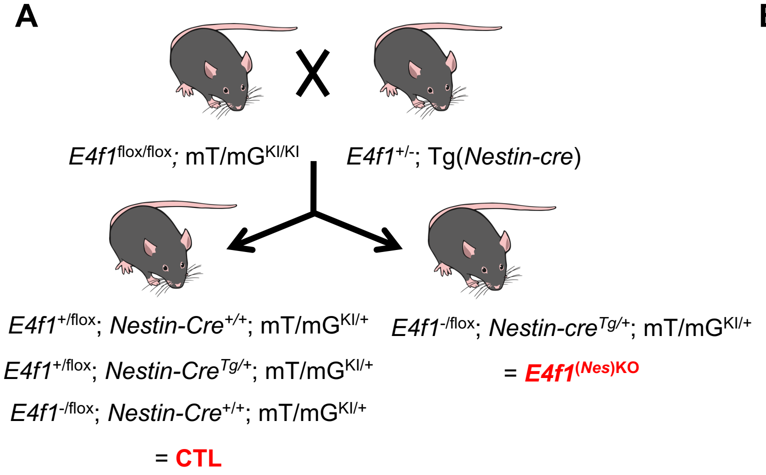

B

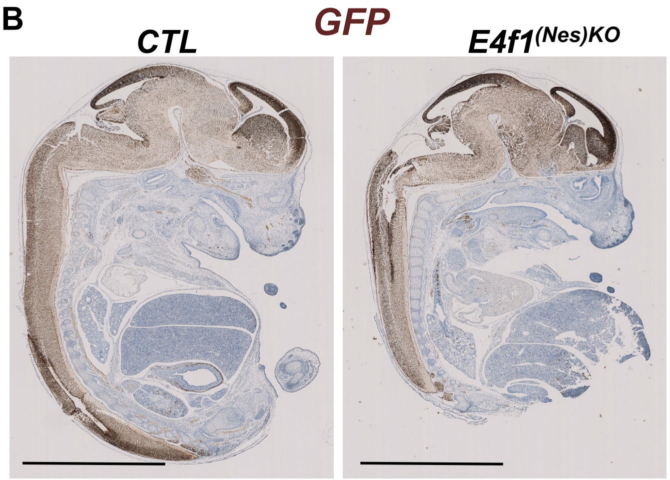

C

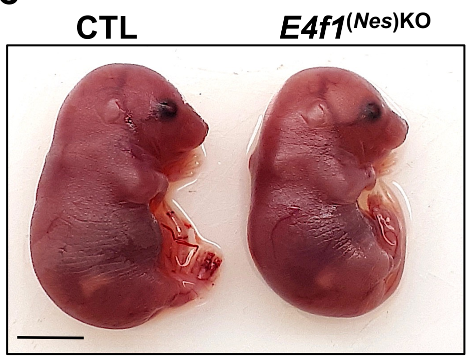

D

		Embryo weight (mg)	p-value	N
E14.5	CTL	315.1 +/- 112.1	ns	42
	E4f1(Nes)KO	320.5 +/- 105.7		42
E16.5	CTL	634.5 +/- 57.2	ns	23
	E4f1(Nes)KO	643.2 +/- 98.7		14
E18.5	CTL	1230.9 +/- 126.2	ns	32
	E4f1(Nes)KO	1299.0 +/- 157.7		22

E

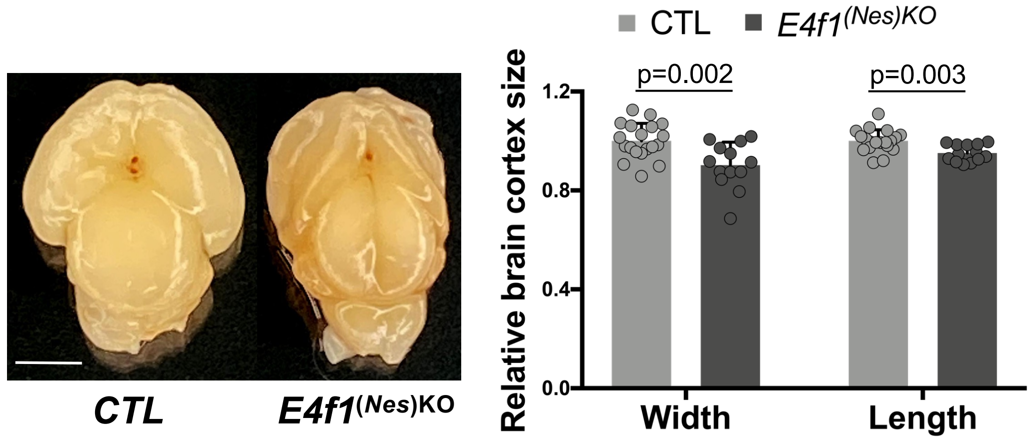

F

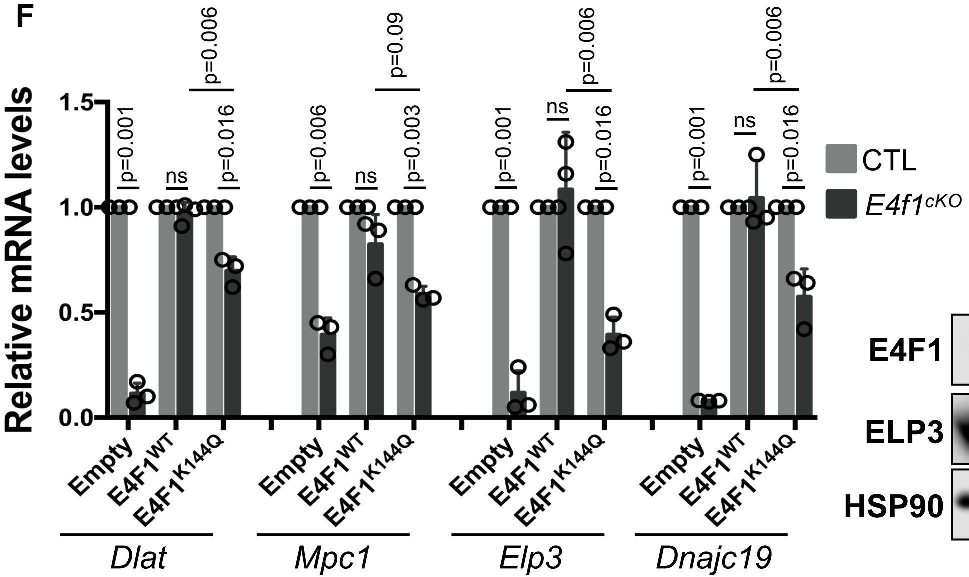

G

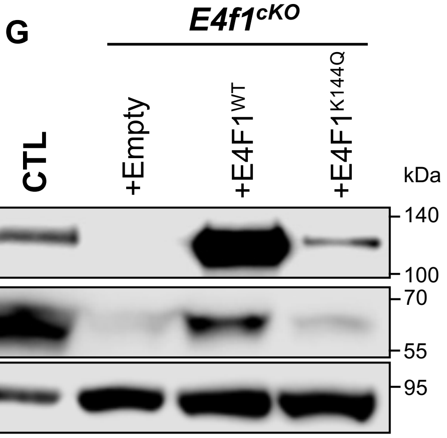

Supplemental Figure S2

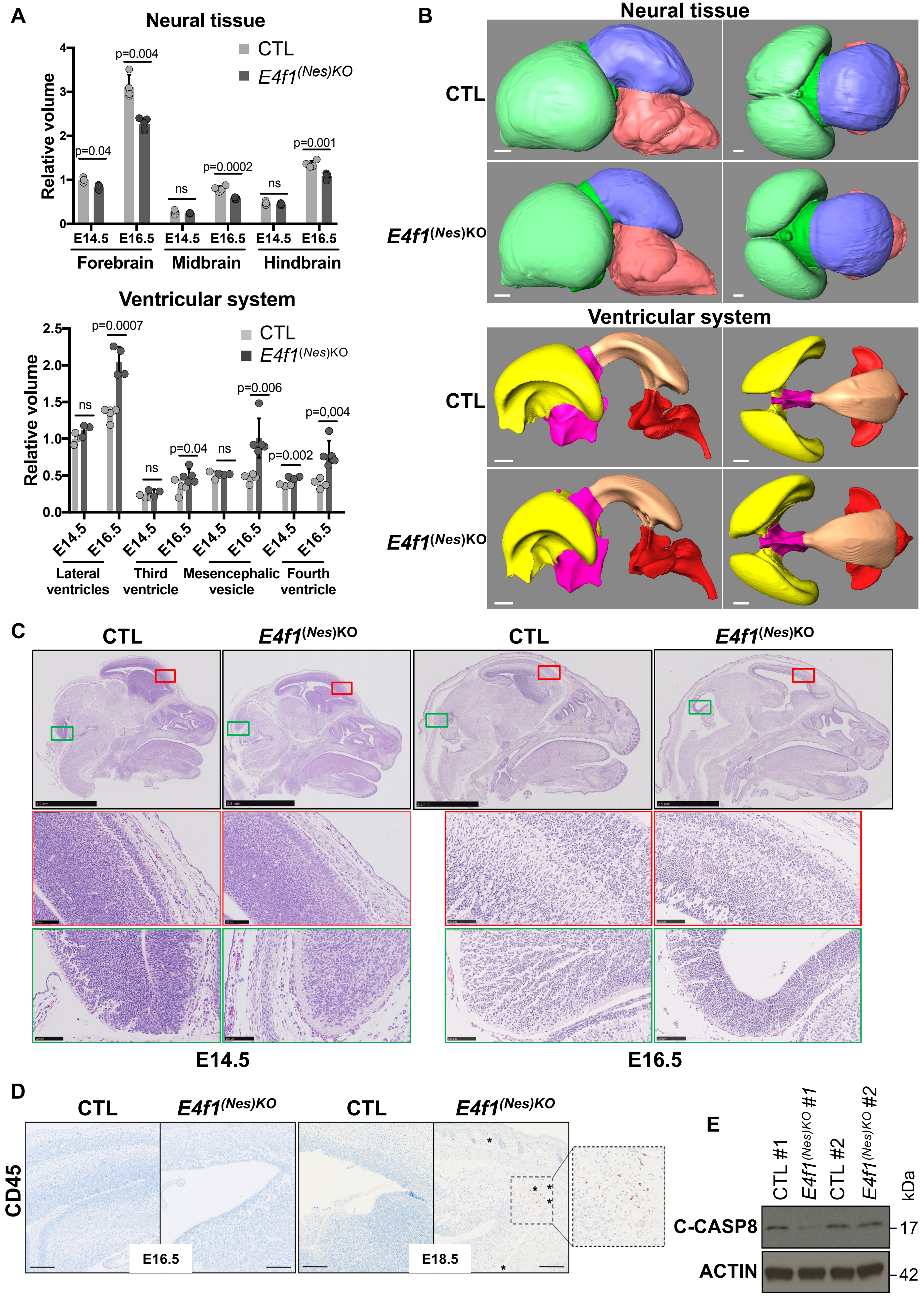

Supplemental Figure S3

A

Gene	FC log2 (*E4f1*^(Nes)KO^ /CTL)	FDR
Elp3	-2.5	2.1E-55
Neurl4	-2.8	6.9E-51
Atf5	2.2	2.3E-38
Dlat	-2.8	5.7E-38
Asns	1.9	2.6E-24
Taz	-2.9	6.0E-24
Wdr7	-2.4	1.5E-23
Sesn2	2.2	1.7E-23
Znhit6	-2.8	6.7E-23
Nsun5	-3.2	2.0E-21
Pbrm1	-1.4	7.3E-21
Chac1	3.0	1.1E-20
Trib3	4.1	1.3E-20
Dnajc19	-3.0	1.2E-19
Fktn	-2.5	2.2E-19
Mrpl15	-2.4	2.7E-19
Eif4ebp1	2.5	2.4E-18
Tti2	-2.1	3.3E-18
Pycr1	1.6	7.1E-18
Leo1	-1.7	2.1E-17

E4F1 direct target genes
ISR/UPR response genes

B

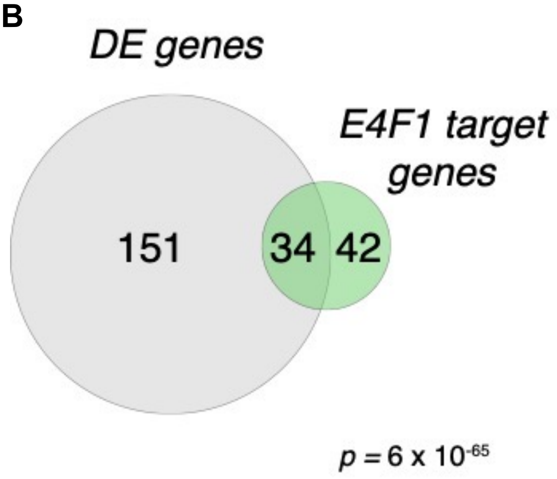

C

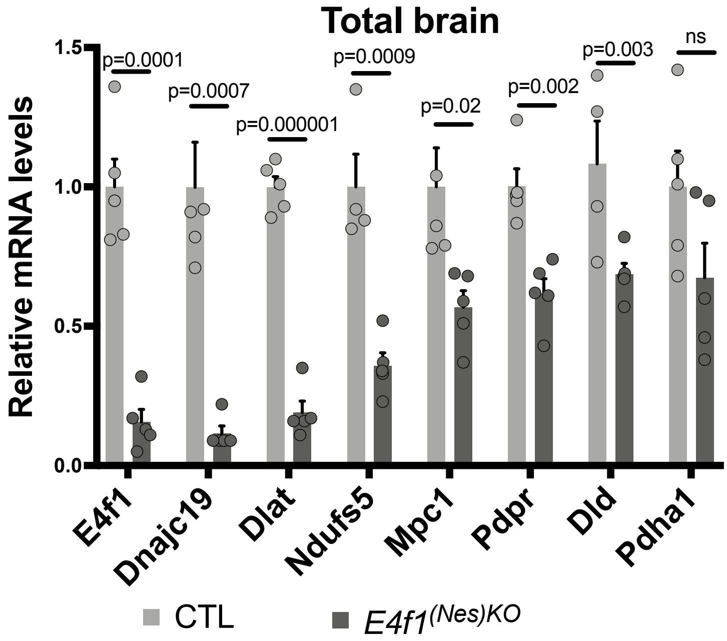

D

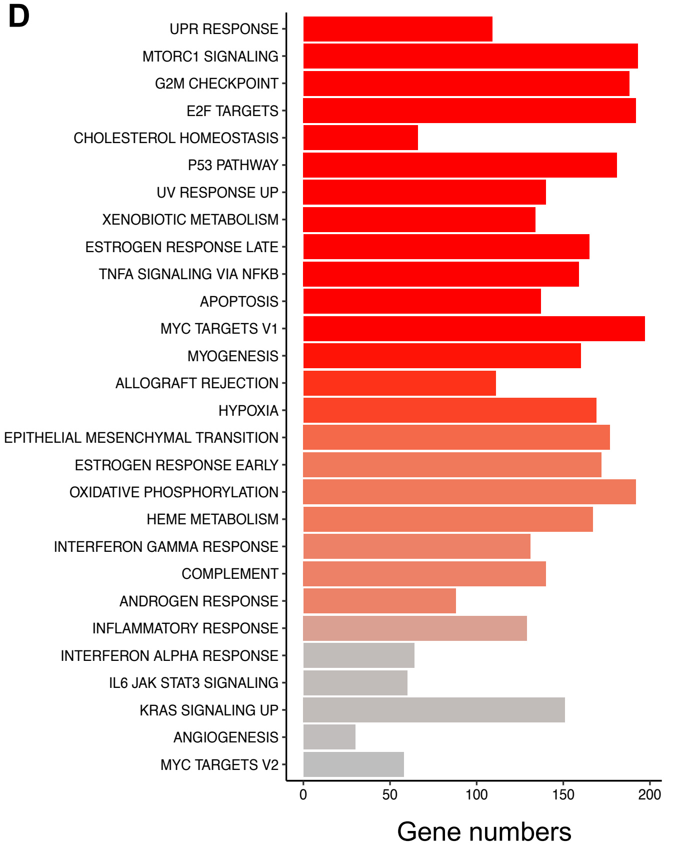

E

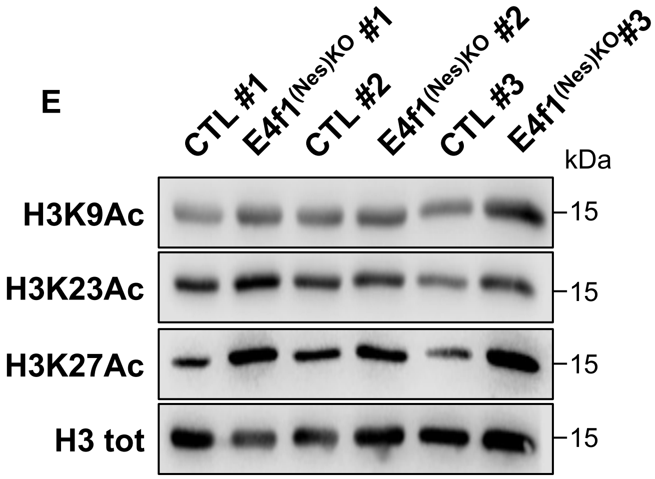

Supplemental Figure S4

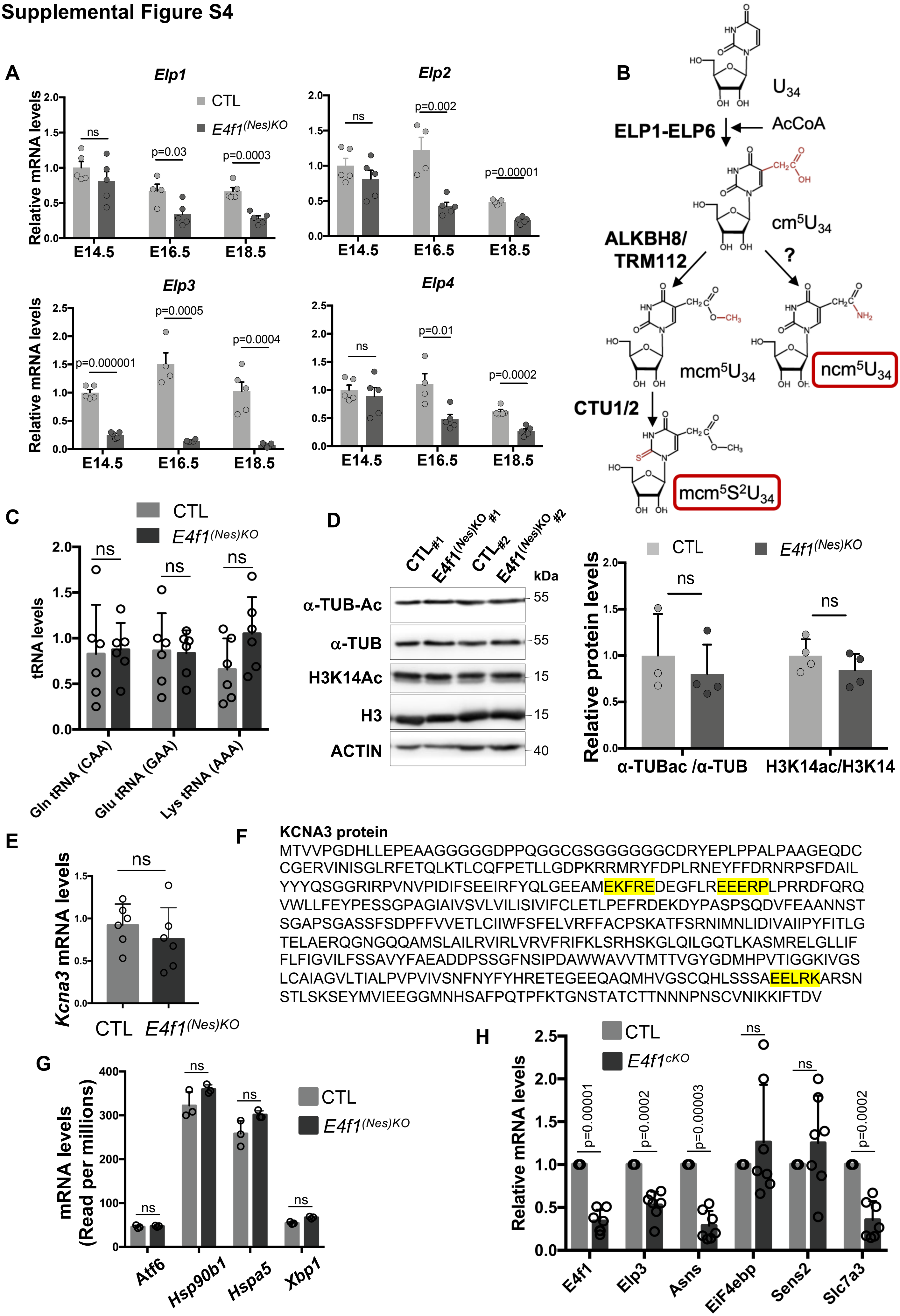

Supplemental Figure S5

A

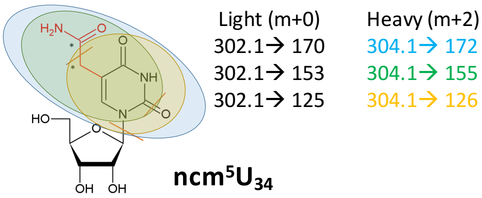

Transition

Light (m+0)	Heavy (m+2)
302.1→ 170	304.1→ 172
302.1→ 153	304.1→ 155
302.1→ 125	304.1→ 126

ncm^{5}U$_{34}$

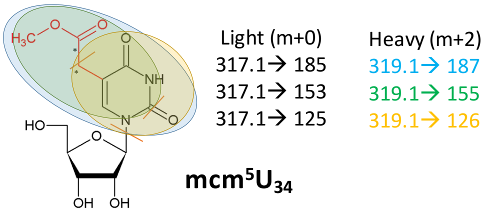

Light (m+0)	Heavy (m+2)
317.1→ 185	319.1→ 187
317.1→ 153	319.1→ 155
317.1→ 125	319.1→ 126

mcm^{5}U$_{34}$

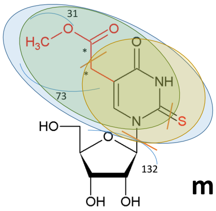

Light (m+0)	Heavy (m+2)
333.1→ 201	335.1→ 203
333.1→ 169	335.1→ 171
333.1→ 141	335.1→ 142

mcm^{5}s^{2}U$_{34}$

B

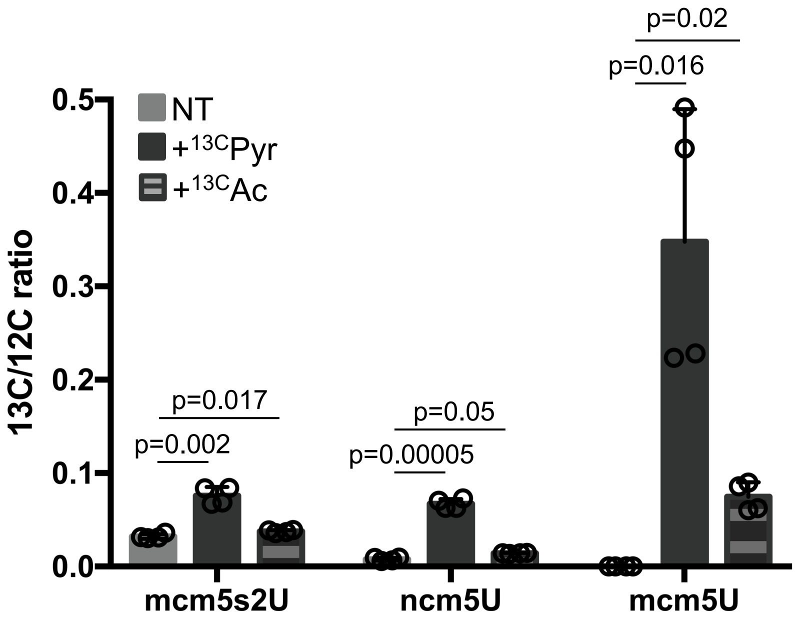

Supplemental Figure S6

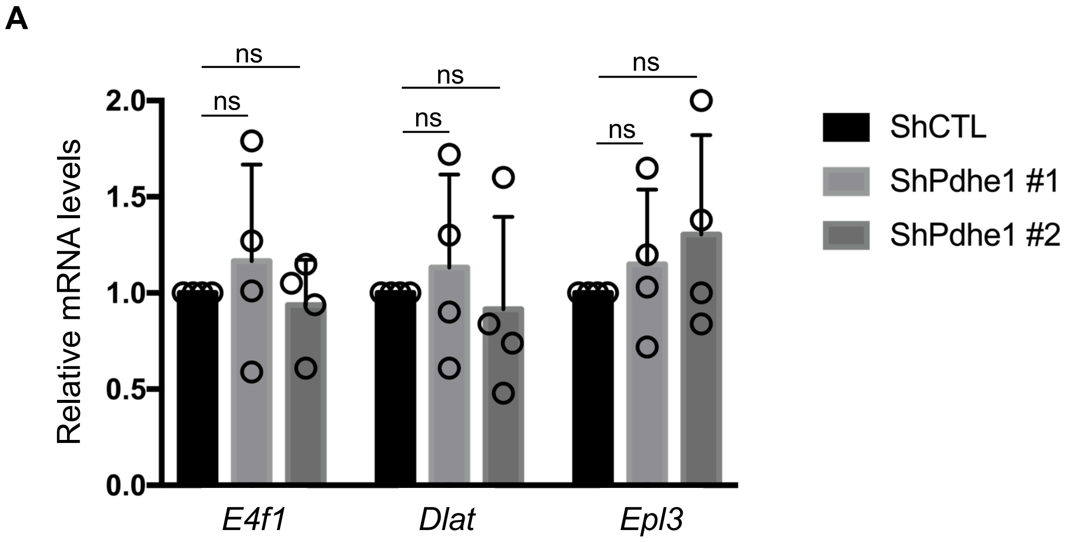

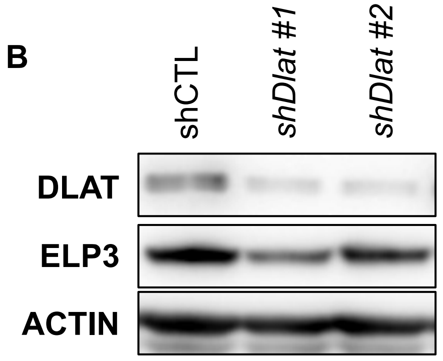

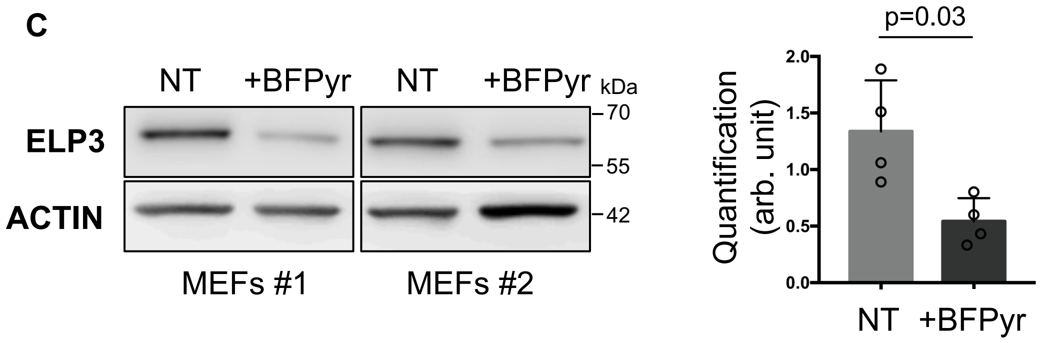

SUPPLEMENTARY INFORMATION

Supplementary Figure S1. E4F1 deficiency in the CNS results in microcephaly. (A) Schematic representation of the crosses used to generate mice lacking E4F1 in the central nervous system (CNS) ("NIAID Visual & Medical Arts. 26/09/2024. Lab Mouse. NIAID NIH BIOART Source. bioart.niaid.nih.gov/bioart/281). (B) Immunohistochemistry (IHC) analysis of GFP protein expression pattern on whole embryo sagittal sections prepared from E14.5 *E4f1*$^{(Nes)KO}$ and CTL littermates. Scale bars, 2.5 mm. Data are representative of experiments performed on n=3 animals/group. (C) Microphotograph of E18.5 *E4f1*$^{(Nes)KO}$ and CTL littermates. Scale bars, 2 mm. (D) Weight of *E4f1*$^{(Nes)KO}$ and CTL embryos at the indicated embryonic developmental stages. N indicates the number of embryos analyzed for each group. (E) Left panel: microphotograph of cortex isolated from E18.5 embryos *E4f1*$^{(Nes)KO}$ and CTL embryos. Scale bar, 500 µm. Right panel: histobars represent the width and the length of the cortex these embryos (n=19 for CTL and n=13 for *E4f1*$^{(Nes)KO}$ embryos). Data were normalized according to each embryo total body length. (F) RT-qPCR analysis of *Dlat, Mpc1*, *Elp3* and *Dnajc19* mRNA levels in *E4f1*^{cKO} and CTL MEFs transduced with lentiviruses encoding E4F1 wild-type (E4F1^{WT}), the E4F1 *K144Q* mutant (E4F1^{K144Q}) or the empty control lentivirus (n=3 independent populations of MEFs/group). Cells were harvested 7 days after Cre-mediated inactivation of *E4f1* and transduction by the indicated lentivirus. (G) Immunoblot analysis of E4F1, ELP3 and HSP90 (loading control) protein levels in *E4f1*^{cKO} and CTL MEFs transduced with lentiviruses encoding E4F1 wild type (E4F1^{WT}), the E4F1 *K144Q* mutant (E4F1^{K144Q}) or the empty control lentivirus. Blots are representative of 3 independent experiments. Data are presented as mean + standard deviation (SD) from the indicated number of animals. Statistical significance was evaluated using unpaired two-sided Student's *t*-test (ns, not significant).

Supplementary Figure S2. *E4f1* inactivation impairs brain development. (A) Analysis of brain lesions in E14.5 and E16.5 *E4f1*$^{(Nes)KO}$ and CTL embryos by High Resolution Episcopic

Microscopy (HREM). Histobars represent the relative volume of different regions of the neuroepithelium tissue and of brain ventricles at E14.5 (n=3 *E4f1*$^{(Nes)KO}$ and CTL embryos) and at E16.5 (n=4 CTL and n=5 *E4f1*$^{(Nes)KO}$ embryos). Data are presented as mean + standard deviation (SD) from the indicated number of animals. Statistical significance was evaluated using unpaired two-sided Student's *t*-test. (B) Representative HREM images showing the 3D volumic reconstruction of different areas of the brain neural tissue (forebrain in green, midbrain in blue, and hindbrain in red) and of the ventricular system (lateral ventricles in yellow, third ventricle in pink, mesencephalic vesicle in orange, fourth ventricle in red) of E14.5 *E4f1*$^{(Nes)KO}$ and CTL littermates (n=3). Scale bars, 175 µm. (C) Hematoxylin and eosin (H&E) -stained sagittal sections prepared from E14.5 and E16.5 *E4f1*$^{(Nes)KO}$ and CTL embryos (n=5). The red and green insets show images at higher magnification of the forebrain and hindbrain of the same embryos, respectively. Scale bars, 2.5 mm and 100 µm for the insets. (D) Immunohistochemistry (IHC) analysis of CD45 protein expression pattern on brain sections prepared from E16.5 and E18.5 *E4f1*$^{(Nes)KO}$ and CTL embryos (n=3). Stars indicate CD45 positive cells. Scale bars, 100 µm. (E) Immunoblot analysis of Cleaved-Caspase 8 (C-CASP8) and HSP90 (loading control) protein levels in E14.5 *E4f1*$^{(Nes)KO}$ and CTL embryos.

Supplementary Figure S3. Gene expression profiling of E4F1-deficient brain. (A) RNA-seq analysis of total brain prepared from E14.5 *E4f1*$^{(Nes)KO}$ and CTL embryos. The list indicates the relative mRNA levels (fold change, log2) of the top 20 differentially expressed genes (DEG) with the most statistically significant False Discovery Rate (FDR-adjusted p-values) identified in E14.5 *E4f1*$^{(Nes)KO}$ embryos. Genes indicated in green and purple are E4F1-direct target genes and ISR-related genes, respectively. (B) Venn diagram showing the overlap between DEG identified in E14.5 *E4f1*$^{(Nes)KO}$ embryos by RNA-seq and the list of E4F1-direct target genes identified in tMEFs, MEFs, and ES cells by ChIP-seq analyses and gene expression profiling (RNA-seq and microarrays). (C) mRNA levels of *E4f1* and of a subset of its direct target genes (*Ndufs5*, *Dnajc19*, *Dlat*, *Mpc1*, *Pdpr*, and *Dld*), or of *Pdha1* (used as a control) were determined by RT-qPCR analysis using total RNAs prepared from total brain of E14.5

E4f1$^{(Nes)KO}$ and CTL embryos (n=5). Data are presented as mean + standard deviation (SD) from the indicated number of animals. Statistical significance was evaluated using unpaired two-sided Student's *t*-test. (D) Pathway enrichment analysis (PEA) of DEG identified in E14.5 *E4f1*$^{(Nes)KO}$ embryos. The size of the histobars represent the number of genes in each category and the color correspond to the indicated adjusted p value. (E) Immunoblot analysis of acetylated histone H3 on lysine 9 (H3K9Ac), lysine 23 (H3K23Ac) and lysine 27 (H3K27Ac) and of total histone H3 (loading control) protein levels in E14.5 *E4f1*$^{(Nes)KO}$ and CTL embryos.

Supplementary Figure S4. *E4f1* controls the Elongator complex and U_{34} codon-biased translation fidelity. (A) RT-qPCR analysis of *Elp1*, *Elp2*, *Elp3*, *Elp4* mRNA levels in total brains of E14.5, E16.5, E18.5 *E4f1*$^{(Nes)KO}$ and CTL embryos (n=5 animals/group). (B) Schematic representation of the cascade leading to U_{34} tRNAs modifications. The enzymes catalyzing the different steps are indicated in bold and the corresponding chemical groups in red. (C) RT-qPCR analysis of Gln (CAA), Glu (GAA) and Lys (AAA) tRNA levels in E14.5, *E4f1*$^{(Nes)KO}$ and CTL embryos (n=6 animals/group). (D) Immunoblot analysis of acetylated α–tubulin (α–TUB-Ac), total α–tubulin (α–TUB), acetylated lysine 14 of histone H3 (H3K14Ac), total histone H3 (H3) and ACTIN (loading control) protein levels in E14.5 *E4f1*$^{(Nes)KO}$ and CTL embryos. Right panel: histobars represent the quantification of immunoblots performed on n=3 CTL and n=4 *E4f1*$^{(Nes)KO}$ independent samples for α–TUB-Ac quantification and n=4 independent embryos of each genotype for H3K14Ac quantification. (E) RT-qPCR analysis of *Kcna3* mRNA levels in E14.5 *E4f1*$^{(Nes)KO}$ and CTL embryos (n=6 animals/group). (F) Penta-Hydrophilic motifs linked to U_{34}-codon dependent translation defects (in yellow) in KCNA3 protein sequence. (G) *Atf6, Hsp90b1, Hspa5* and *Xbp1* mRNA levels in E14.5 *E4f1*$^{(Nes)KO}$ and CTL embryos measured by RNA-seq (n=3 animals/group). (H) Relative mRNA levels of genes related to the ISR response in *E4f1*^{cKO} and CTL MEFs cells determined by RT-qPCR (n=6 independent populations of cells/group). Data are presented as mean + standard error of the mean (SEM) for Fig. S4A or mean + standard deviation (SD) for Fig. S4C-F from the indicated

number of animals. Statistical significance was evaluated using unpaired two-sided Student's *t*-test (ns, not significant).

Supplementary Figure S5. U_{34} tRNAs acetylation by pyruvate derived AcCoA. (A) Schematic representation of the different transitions (indicated in yellow, green and blue) and their respective molecular weight of the m+2 isotopologues of the indicated U_{34} tRNA modifications detected by LC-MS/MS upon incubation with [U-^{13}C]-pyruvate. (B) Stable isotope tracing experiments in MEFs cultured for the last 6 hours in the presence of [U-^{13}C]-pyruvate or acetate. ^{13}C-enrichment in $mcm^5s^2U_{34}$, ncm^5U_{34} and mcm^5U_{34} was determined by LC-MS/MS in 4 independent replicates for each condition. Data are represented as the relative ratio between the ^{13}C-labelled and unlabelled U_{34} modifications. Data are presented as mean + standard deviation (SD) from the indicated number of samples. Statistical significance was evaluated using unpaired two-sided Student's *t*-test.

Supplementary Figure S6. Crosstalk between PDC and the Elongator complex. (A) RT-qPCR analysis of *E4f1*, *Dlat* and *Elp3* mRNA levels in MEFs transduced with lentiviruses encoding 2 different shRNAs targeting *Pdhe1* or a control shRNA (shCTL) (n=4 independent experiments). (B) Representative immunoblots showing DLAT, EPL3 and ACTIN (loading control) protein levels in MEFs transduced with lentiviruses encoding 2 different shRNAs targeting *Dlat* or a control shRNA (shCTL) (n=4 independent experiments). (C) Left panel: Immunoblot analysis of EPL3 and ACTIN (loading control) protein levels in MEFs treated with Beta-FluoroPyruvate (BFPyr) for 24 hrs. Right panel: histobars represent the quantification of immunoblots performed on 4 independent population of MEFs.

Supplementary Table 1

List of oligonucleotides used in this study.

Supplementary Table 1

	Application	Forward	Reverse
E4f1WT/Flox	Genotyping	CCCCAAGAAGCCCAAGTTCCTGAT	CCTCTGTTCCACATACACTTCATTC
E4f1KO	Genotyping	CACTGCCTTGGAGGACTTTGTCCA	GGCTGCTGCGTGGATTTC
Nestin-Cre	Genotyping	CGGTCGATGCAACGAGTGATGAGG	CCAGAGACGGAAATCCATCGCTCG
Rosa26-CreER	Genotyping	CGATGCAACGAGTGATGAGGTTC	GCACGTTCACCGGCATCAAC
E4f1	RT-qPCR	CCAAAGCCTACCTGCTCAAG	CTGGGCATTCTTGGTTTTGT
Ndufs5	RT-qPCR	TAAGCGCAGAACAGCCCTAT	TCATTAGCTTCTCCCGCTGT
Dnajc19	RT-qPCR	GTAGCAGTCGGGTTGACCAT	TTGGCAGTAGGGCTTACACC
Taz	RT-qPCR	AGGAAGCGTCCTTGCTTGTT	GCGTCCTTGCCTCACCTTTT
Wdr7	RT-qPCR	ACCCAGTCCCAGCAGAGTAT	AGTGAGGTGTTCATCTGCCA
Neurl4	RT-qPCR	CCATGACCAATTTACGCTCTGGG	GATCAACGACAGCATAGACACCC
Dlat	RT-qPCR	TTGGCCTGTCTGAAAGTTCC	TTACCCTCTCTCGCTTTGGA
Dld	RT-qPCR	CCTTGTAGCTACGGGCTCAG	CCCACATGACCCAAAAATTC
Mpc1	RT-qPCR	TCCAGAGATTATCAGTGGGCGGAT	GCCAGTTTCGAGGTTGTACCTTGT
Pdpr	RT-qPCR	AAGACAAAGGACTAGCCCAGC	GATAGGCCACGGATGTACCC
Pdha1	RT-qPCR	GCTGGTTGCTTCCCGTAAT	TAGTACTTGAGCCCATCCTCTC
Elp1	RT-qPCR	TGTTTCTCGTCTCCCGTGTG	AGCAATTTCTACGACGCCCT
Elp2	RT-qPCR	GCTGGAGGTGTCTCATGTGTT	GCACAACGGAGCAGGATGT
Elp3	RT-qPCR	GGGAGGAAGTGGATTCTCTG	ACGTCTTTCCCCTGCTCAT
Elp4	RT-qPCR	GGCGGCACCTCTAGTTTCC	AGCAGTTGTCCATTCCGCA
Trib3	RT-qPCR	AGCACTTTAGCAGCGGAAGA	AGGTGTAGCTCGCATCTTGT
Chac1	RT-qPCR	CTTGAAGACCGTGAGGGCTG	GGCTTCCAGGTGCTCATCTT
Slc7a3	RT-qPCR	TCCAGATTTCTTTGCCTTGG	CATCCCTGTGAACACTTTGG
Eif4ebp	RT-qPCR	GGGGACTACAGCACCACTCC	GTTTTGGCCACAGGTGAGTT
Asns	RT-qPCR	AAGGGCCTGACTCCATAGGT	TACAACCACAAGGGGCTACA
Atf5	RT-qPCR	GGCCCCTATGAGGTCCTTG	CGCTCAGTCATCCAATCAGA
Sesn2	RT-qPCR	GCATTACCTGCTGCTGCATA	GCAGGAACTCAGTCATGTGG
Slc6a9	RT-qPCR	TCTCCTGGGTGGTTGTCTTC	ACAGAATGGTCAGCACCACA
Gadd34	RT-qPCR	GACTCAAGCCAGAGTCCCTG	TAGAGGAATCTCGGGGTCCT
Atf4	RT-qPCR	AATGGCCGGCTATGG ATGAT	CAATCTGTCCCGGAAAAGGC
Nars	RT-qPCR	TGTGGCACAGAGAACAGATGA	TTTTTGCTTCCTCCAGGTTC
Chop	RT-qPCR	CCCCAGGAAACGAAGAGGAA	TGACCTCTGTTGGCCCTG
Cars	RT-qPCR	TATCCTGAGGAGGGTGCTGA	TTTCTGCTCCCGATACTGCT
Xbp1	RT-qPCR	TGTCACCTCCCCAGAACATC	CCAACTTGTCCAGAATGCCC
Xbp1-s	RT-qPCR	GGAATGGACACGCTGGATCC	GCCTGCACCTGCTGCGGA
Xbp1-u	RT-qPCR	AAGAACACGCTTGGGAATGG	GCACATAGTCTGAGTGCTGC
Kcna3	RT-qPCR	AGGCGGGATAGTCTTTCTCATC	GGGGCATTGCCATTGTGTC
Elp3	ChIP	CCCAGACCAAGCAGTAATTCCCAT	AAGTTTCTCCGCCTTCTAAACCGCC
