# Supplementary material for: E4F1 coordinates pyruvate metabolism and the activity of the elongator complex to ensure translation fidelity during brain development
Source: Nat Commun. 2025 Jan 2;16:67. doi: 10.1038/s41467-024-55444-y (PMC11696611; doi:10.1038/s41467-024-55444-y)
Supplement: Supplementary file 2 — Description of Additional Supplementary Files [file 41467_2024_55444_MOESM2_ESM.pdf]

Description of Additional Supplementary Files

Supplementary Data 1

Differential expression analysis based on RNA-seq data generated for the brain of E14.5 E4f1(Nes)KO vs CTL embryos. Differentially expressed genes (DEG) were analyzed using likelihood ratio tests (LRT) - FDR-adjusted p-values.

Supplementary Data 2

E4F1 core transcriptional program. List of E4F1 direct target genes defined by the presence of an E4F1 ChIP-seq peak in tMEFs or mES cells, and showing differential mRNA levels between E4f1cKO and CTL tMEFs, E4f1cKO vs CTL MEFs, and between the brain of E14.5 E4f1(Nes)KO and CTL embryos.

Supplementary Data 3

Polysome profiling analysis of total brains prepared from E14.5 E4f1(Nes)KO and CTL embryos. For statistical analysis, datasets from polysome fractions (light, heavy, and total) were analyzed independently using R v4.4.0. Differential expression (DE) analysis for each fraction was conducted with edgeR v4.2.1, comparing gene expression between E4f1(Nes)KO and CTL animals. After estimating the signal dispersion, the abundance of transcripts in a given fraction was considered significantly different in likelihood ratio tests when exhibiting an absolute foldchange ≥ 1.2 and a FDR-adjusted p-value < 0.2.
