# Supplementary material for: E4F1 coordinates pyruvate metabolism and the activity of the elongator complex to ensure translation fidelity during brain development
Source: Nat Commun. 2025 Jan 2;16:67. doi: 10.1038/s41467-024-55444-y (PMC11696611; doi:10.1038/s41467-024-55444-y)
Supplement: Supplementary file 6 — Reporting Summary [file 41467_2024_55444_MOESM6_ESM.pdf]

nature portfolio

Corresponding author(s): Laurent Le Cam

Last updated by author(s): Sep 1, 2024

Reporting Summary

Nature Portfolio wishes to improve the reproducibility of the work that we publish. This form provides structure for consistency and transparency in reporting. For further information on Nature Portfolio policies, see our Editorial Policies and the Editorial Policy Checklist.

Statistics

For all statistical analyses, confirm that the following items are present in the figure legend, table legend, main text, or Methods section.

n/a	Confirmed	
☐	☒	The exact sample size (*n*) for each experimental group/condition, given as a discrete number and unit of measurement
☐	☒	A statement on whether measurements were taken from distinct samples or whether the same sample was measured repeatedly
☐	☒	The statistical test(s) used AND whether they are one- or two-sided<br>*Only common tests should be described solely by name; describe more complex techniques in the Methods section.*
☐	☒	A description of all covariates tested
☐	☒	A description of any assumptions or corrections, such as tests of normality and adjustment for multiple comparisons
☐	☒	A full description of the statistical parameters including central tendency (e.g. means) or other basic estimates (e.g. regression coefficient) AND variation (e.g. standard deviation) or associated estimates of uncertainty (e.g. confidence intervals)
☐	☒	For null hypothesis testing, the test statistic (e.g. *F*, *t*, *r*) with confidence intervals, effect sizes, degrees of freedom and *P* value noted<br>*Give P values as exact values whenever suitable.*
☒	☐	For Bayesian analysis, information on the choice of priors and Markov chain Monte Carlo settings
☒	☐	For hierarchical and complex designs, identification of the appropriate level for tests and full reporting of outcomes
☒	☐	Estimates of effect sizes (e.g. Cohen's *d*, Pearson's *r*), indicating how they were calculated

Our web collection on statistics for biologists contains articles on many of the points above.

Software and code

Policy information about availability of computer code

Data collection	No public datasets were analyzed in this study

Data analysis	For RNA-sequencing, STAR v2.5.3a was used to align reads on the reference genome mm10. Gene expression was determined with RSEM 1.2.28 on RefSeq catalogue, prior to normalisation and differential analysis with DESeq2 bioconductor package. Multiple hypothesis adjusted p-values were calculated with the Benjamini-Hochberg procedure to control FDR. GSEA and PEA were performed with fgsea bioconductor package on Hallmark gene sets from MSigDB collections, completed with some custom gene sets.<br>Statistical significance was evaluated using unpaired bilateral Student's t-test with Microsoft Excel vs 15.33 and BiostatTGV website (https://biostatgv.sentiweb.fr/).<br>Polysome profiling datasets were pre-processed using the nf-core RNA-seq pipeline v3.11.044. Initial quality control of raw reads was conducted with FastQC v0.11.9. Trim Galore v0.6.7 was used for reads trimming. Reads were aligned to the mouse reference genome (GRCm38) using STAR v2.7.9a. Transcript-level quantification was performed using Salmon v1.10.1, and gene-level summarization was carried out using tximport v1.32.0. Post-alignment quality was assessed with RSeQC v3.0.1 to ensure data integrity and alignment accuracy. For statistical analysis, datasets from polysome fractions (light, heavy, and total) were analyzed independently using R v4.4.0. Differential expression (DE) analysis for each fraction was conducted with edgeR v4.2.1. Codon usage bias was assessed by comparing codon frequencies in transcripts controlled at the post-trancriptional level in E4F1-deficient cells to those in transcripts from the entire GRCm38 transcriptome using $\chi 2$ tests. In the corresponding proteins, enrichment of the specific hydrophilic motif linked to U34-dependent translational defects was conducted using the AME (Analysis of Motif Enrichment) tool from the MEME suite v1.12.0, using the occurrence of this motif in the full GRCm38 proteome as a reference.<br>ImageJ 1.50i and Fiji 2.3.0 were used to crop and quantify images from unprocessed images.<br>For schematic representation, we used NIH BIOART Source (https://bioart.niaid.nih.gov/) and BioRender (https://www.biorender.com/).

For manuscripts utilizing custom algorithms or software that are central to the research but not yet described in published literature, software must be made available to editors and reviewers. We strongly encourage code deposition in a community repository (e.g. GitHub). See the Nature Portfolio guidelines for submitting code & software for further information.

Data

Policy information about availability of data

All manuscripts must include a data availability statement. This statement should provide the following information, where applicable:

- Accession codes, unique identifiers, or web links for publicly available datasets
- A description of any restrictions on data availability
- For clinical datasets or third party data, please ensure that the statement adheres to our policy

RNA-seq and polysome profiling datasets are available on Gene Expression Omnibus with the accessions GSE158595 (https://www.ncbi.nlm.nih.gov/geo/query/acc.cgi?acc=GSE158595) & GSE278444 (https://www.ncbi.nlm.nih.gov/geo/query/acc.cgi?acc=GSE278444).
The source data related (uncroped gels and raw data) to Figures 1-8 and Supplementary Figures 1-6 is provided as a Source Data file. All data that support the findings of this study are available within the article, its Supplementary Information and the publicly accessible GEO repository.

Research involving human participants, their data, or biological material

Policy information about studies with human participants or human data. See also policy information about sex, gender (identity/presentation), and sexual orientation and race, ethnicity and racism.

Reporting on sex and gender	The sex of the patients was not considered in this study. All experiments were conducted on fibroblasts isolated from both male and female Leigh syndrome patients and control individuals.
Reporting on race, ethnicity, or other socially relevant groupings	There is no socially relevant categorization variable used in this study.
Population characteristics	Skin fibroblasts were isolated from Leigh syndrome patients and age matched control individuals between 1 month and 5 years of age.
Recruitment	Leigh syndrome patients or control individuals were recruited at the Kremlin Bicêtre hospital (Paris, FRANCE, agreement number DC 2009-939 from the "Comité d'Ethique de L'hopital Bicêtre Assistance Publique Hopitaux de Paris") or at the Foundation IRCSS Institute of Neurology Carlo Besta (Milan, ITALY, agreement number GGP15041 from "Comitato Etico Regione Lombardia") by clinicians involved in this study (Lebigot Elise, Legati Andrea and Ghezzi Daniele). Leigh syndrome patients were categorized in PDH- or Electron Transport Chain (ETC)- deficient according to the detection of previously described pathogenic variants linked to the LS determined by PCR or based on their E4F1 status by whole genome sequencing.
Ethics oversight	Patients were recruited after they signed a written informed consent authorizing their participation in the study and publication of personal clinical information after anonymization.

Note that full information on the approval of the study protocol must also be provided in the manuscript.

Field-specific reporting

Please select the one below that is the best fit for your research. If you are not sure, read the appropriate sections before making your selection.

☒ Life sciences ☐ Behavioural & social sciences ☐ Ecological, evolutionary & environmental sciences

For a reference copy of the document with all sections, see nature.com/documents/nr-reporting-summary-flat.pdf

Life sciences study design

All studies must disclose on these points even when the disclosure is negative.

Sample size	For in vivo experiments, the size of the experimental groups was determined using the G*power software based on previous comparable studies from our team and pilot studies which allowed us to predict the expected biological variability (Lacroix et al., PNAS, 2016; Goguet-Rubio et al., PNAS, 2016; Lacroix et al., Nat Comms, 2021). The size of the experimental groups were defined to reach statistical significance with an alpha value of 0,05.<br>For in vitro experiements, no statistical method was used to define sample size but all were realized on 3 to 6 independent populations of primary cells of a given genotype as indicated in the figure legends.
Data exclusions	No data were excluded from the analyses.
Replication	All in vivo analyses were performed on a sufficient number of animals of the same genotype to reach statistical significance with a minimum of 5 animals per group. Data represented these results as the mean + standard deviation as indicated on the figures .<br>In vitro analyses performed on primary cells were done on 3 to 6 independent experiments (replicate information is indicated in each figure legends).
Randomization	For in vivo studies, pregnante females were randomized for vehicule or ISRIB treatment. Embryos were collected blindly and assigned to experimental groups after genotyping.
Blinding	For in vivo experiments and histological analysis, investigators were blinded to the identities (E4F1 and Cre genotypes), which were revealed only after data collection.<br>In vitro experiments (RT-qPCR and immunoblots) were not blinded since the same investigators performed the experiment and the analysis.

Behavioural & social sciences study design

All studies must disclose on these points even when the disclosure is negative.

Study description	*Briefly describe the study type including whether data are quantitative, qualitative, or mixed-methods (e.g. qualitative cross-sectional, quantitative experimental, mixed-methods case study).*
Research sample	*State the research sample (e.g. Harvard university undergraduates, villagers in rural India) and provide relevant demographic information (e.g. age, sex) and indicate whether the sample is representative. Provide a rationale for the study sample chosen. For studies involving existing datasets, please describe the dataset and source.*
Sampling strategy	*Describe the sampling procedure (e.g. random, snowball, stratified, convenience). Describe the statistical methods that were used to predetermine sample size OR if no sample-size calculation was performed, describe how sample sizes were chosen and provide a rationale for why these sample sizes are sufficient. For qualitative data, please indicate whether data saturation was considered, and what criteria were used to decide that no further sampling was needed.*
Data collection	*Provide details about the data collection procedure, including the instruments or devices used to record the data (e.g. pen and paper, computer, eye tracker, video or audio equipment) whether anyone was present besides the participant(s) and the researcher, and whether the researcher was blind to experimental condition and/or the study hypothesis during data collection.*
Timing	*Indicate the start and stop dates of data collection. If there is a gap between collection periods, state the dates for each sample cohort.*
Data exclusions	*If no data were excluded from the analyses, state so OR if data were excluded, provide the exact number of exclusions and the rationale behind them, indicating whether exclusion criteria were pre-established.*
Non-participation	*State how many participants dropped out/declined participation and the reason(s) given OR provide response rate OR state that no participants dropped out/declined participation.*
Randomization	*If participants were not allocated into experimental groups, state so OR describe how participants were allocated to groups, and if allocation was not random, describe how covariates were controlled.*

Ecological, evolutionary & environmental sciences study design

All studies must disclose on these points even when the disclosure is negative.

Study description	*Briefly describe the study. For quantitative data include treatment factors and interactions, design structure (e.g. factorial, nested, hierarchical), nature and number of experimental units and replicates.*
Research sample	*Describe the research sample (e.g. a group of tagged Passer domesticus, all Stenocereus thurberi within Organ Pipe Cactus National Monument), and provide a rationale for the sample choice. When relevant, describe the organism taxa, source, sex, age range and any manipulations. State what population the sample is meant to represent when applicable. For studies involving existing datasets, describe the data and its source.*

Sampling strategy	*Note the sampling procedure. Describe the statistical methods that were used to predetermine sample size OR if no sample-size calculation was performed, describe how sample sizes were chosen and provide a rationale for why these sample sizes are sufficient.*
Data collection	*Describe the data collection procedure, including who recorded the data and how.*
Timing and spatial scale	*Indicate the start and stop dates of data collection, noting the frequency and periodicity of sampling and providing a rationale for these choices. If there is a gap between collection periods, state the dates for each sample cohort. Specify the spatial scale from which the data are taken*
Data exclusions	*If no data were excluded from the analyses, state so OR if data were excluded, describe the exclusions and the rationale behind them, indicating whether exclusion criteria were pre-established.*
Reproducibility	*Describe the measures taken to verify the reproducibility of experimental findings. For each experiment, note whether any attempts to repeat the experiment failed OR state that all attempts to repeat the experiment were successful.*
Randomization	*Describe how samples/organisms/participants were allocated into groups. If allocation was not random, describe how covariates were controlled. If this is not relevant to your study, explain why.*
Blinding	*Describe the extent of blinding used during data acquisition and analysis. If blinding was not possible, describe why OR explain why blinding was not relevant to your study.*

Did the study involve field work? ☐ Yes ☐ No

Field work, collection and transport

Field conditions	*Describe the study conditions for field work, providing relevant parameters (e.g. temperature, rainfall).*
Location	*State the location of the sampling or experiment, providing relevant parameters (e.g. latitude and longitude, elevation, water depth).*
Access & import/export	*Describe the efforts you have made to access habitats and to collect and import/export your samples in a responsible manner and in compliance with local, national and international laws, noting any permits that were obtained (give the name of the issuing authority, the date of issue, and any identifying information).*
Disturbance	*Describe any disturbance caused by the study and how it was minimized.*

Reporting for specific materials, systems and methods

We require information from authors about some types of materials, experimental systems and methods used in many studies. Here, indicate whether each material, system or method listed is relevant to your study. If you are not sure if a list item applies to your research, read the appropriate section before selecting a response.

Materials & experimental systems

n/a	Involved in the study	
☐	☒	Antibodies
☐	☒	Eukaryotic cell lines
☒	☐	Palaeontology and archaeology
☐	☒	Animals and other organisms
☒	☐	Clinical data
☒	☐	Dual use research of concern
☒	☐	Plants

Methods

n/a	Involved in the study	
☒	☐	ChIP-seq
☒	☐	Flow cytometry
☒	☐	MRI-based neuroimaging

Antibodies

Antibodies used

For Western blotting:
E4F1 (1/1000) (Fajas et al. 2000, 1/2000)
C-CASP3 (cl D175, Cell Signaling, 9661S, lot 43, 1/1000)
DLAT (Santa Cruz, sc-271534, lot C1813, 1/500)
MPC1 (Sigma, HPA045119, lot E105936, 1/1000)
DLD (cl G2, Santa Cruz, sc-365977, lot F1611, 1/1000)
PDHE1 (9H9AF5, Life Technologies, 459400, lot J4161, 1/500)
ELP3 (Abcam, ab190907, lot GR3180892-3, 1/500)
ELP1 (Abcam, ab62498, lot GR3369590, 1/500)
TUBULIN (Sigma, T6199, lot 115M4796V, 1/3000)

ACTIN (Sigma, A3854, lot 100M4782, 1/7000)
Histone H3K9Ac (Cell Signaling, 9649S, lot 13, 1/1000)
Histone H3K23Ac (Abcam, ab61234, lot GR82698-4, 1/1000)
Histone H3K27Ac (Abcam, ab4729, lot 17, 1/1000)
total Histone H3 (cl D1H2, Cell Signaling, 4499S, lot 9, 1/1000)
Histone H3 K14Ac (clD4B9, Cell Signaling, 7627, 1/1000)
Acetylated TUBULIN (sc23950, lot E2218, 1/500)
HSP90 (13988S, Cell Signaling, lot 2, 1/1000)
KCNA3 (Diagomics, AF6702, Lot 23, 1/500)
Cleaved Caspase 8 (Proteintech, 66093-1, lot 10023946, 1/1000)
For Immunohistochemistry/Immunofluorescence:
GFP (Invitrogen, A10262, lot 2260907, 1/100)
Cleaved-CASP3 (cl D175, Cell Signaling, 9661S, lot 43, 1/100)
CD45 (Biosciences, 14-051-82, lot 4316815, 1/500)
MCT4 (Proteintech, 22787-1-AP, lot 00049309, 1/200)
ATF4 (cl D4B8, Cell Signaling, 11815, lot 6, 1/200)
phospho-eIF2a (Cliniscience, P04387, lot P04387, 1/500)
Ki67 (Abcam, ab15580, lot GR3452706-1, 1/500)
SOX2 (Santa Cruz, sc17320, lot #H0516, 1/500)
TBR2 (eBiosciences, 14-4875-82, lot 2504948, 1/250)
TBR1 (Abcam, ab31940, lot GR3182037-1, 1/500)
GFAP (Aves Labs, AB2313547, lot 4937976, 1/200)
NeuN (cl IB7, Biolegend, 834501, lot B352650, 1/500)

Validation

Polyclonal anti E4F1 antibody used in ChIP experiments was validated in previous studies (Fajas et al., PNAS, 2000; Lacroix et al., Nat Comms, 2021).
The specificity of the anti-PDHE1 antibody was validated using cellular extracts prepared from cells expressing shRNAs against Pdha1 .
The specificity of the anti-ELP3 antibody was validated using cellular extracts prepared from cells expressing shRNAs against Elp3.
All other antibodies were validated by the corresponding manufacturers.

Eukaryotic cell lines

Policy information about cell lines and Sex and Gender in Research

Cell line source(s)

Mouse embryonic fibroblasts (Mefs) were isolated from E13.5 embryos and included in experimental groups according to their genotype.
Populations of primary neurons and glial cells were isolated from P1 pups and included in experimental groups according to their genotype.
The HEK-293T cells were used to produce lentiviral and retroviral particles.
Human skin fibroblasts were isolated from Leigh syndrome patients or age match control individuals.
N2A murine neuroblastoma cell line was used for ChIP experiments.

Authentication

Cell line authentication is not relevant for primary MEFs and primary populations of neurons/glial cells and human skin fibroblasts.
The HEK293T and N2A cells (provided by Nguyen Lab) were not authentified.

Mycoplasma contamination

All cells were tested negative for Mycoplasma contamination using a commercial kit (Lonza, #LT07-318).

Commonly misidentified lines
(See ICLAC register)

Not relevant with the present study.

Palaeontology and Archaeology

Specimen provenance

Provide provenance information for specimens and describe permits that were obtained for the work (including the name of the issuing authority, the date of issue, and any identifying information). Permits should encompass collection and, where applicable, export.

Specimen deposition

Indicate where the specimens have been deposited to permit free access by other researchers.

Dating methods

If new dates are provided, describe how they were obtained (e.g. collection, storage, sample pretreatment and measurement), where they were obtained (i.e. lab name), the calibration program and the protocol for quality assurance OR state that no new dates are provided.

☐ Tick this box to confirm that the raw and calibrated dates are available in the paper or in Supplementary Information.

Ethics oversight

Identify the organization(s) that approved or provided guidance on the study protocol, OR state that no ethical approval or guidance was required and explain why not.

Note that full information on the approval of the study protocol must also be provided in the manuscript.

Animals and other research organisms

Policy information about studies involving animals; ARRIVE guidelines recommended for reporting animal research, and Sex and Gender in Research

Laboratory animals	The following strains were used in this study: E4f1tm1.1Llca, E4f1tm1Pisc, Tg(Nes-cre)1Kln, Gt(ROSA)26Sortm1(cre/ERT2)Tyj (Le Cam et al., 2004; Lacroix et al., 2010; Tronche et al., 1999, Muzumdar et al., 2007). Mice were interbred and maintained on a mix 129Sv/J; C57Bl/6J background and were housed in a pathogen free barrier facility (room temperature 22°C; relative humidity 55%, and a 12-h-light–dark cycle). Mice were maintained under chow (A03, Safe) containing 22 kcal% protein, 65 kcal% carbohydrate and 13 kcal% fat. Pregnant females were between 8 and 16 weeks of age. Embryos were studied at E14.5, E16.5, E18.5 of embryonic development, and newborns at P1.
Wild animals	The study did not involved wild animals.
Reporting on sex	Both males and females embryos were used in this study.
Field-collected samples	No field collected samples were used in the study.
Ethics oversight	All procedures were approved by the ethic committee for animal warefare of the region Languedoc Roussillon (Comité d'Ethique en Expérimentation Animal Languedoc-Roussillon), an accredited institution of the French Minister for Education, Research and Innovation (agreement number #18030-201812111250227). Animal housing and euthanasia were performed in accordance to the 3R rules.

Note that full information on the approval of the study protocol must also be provided in the manuscript.

Clinical data

Policy information about clinical studies

All manuscripts should comply with the ICMJEguidelines for publication of clinical research and a completedCONSORT checklist must be included with all submissions.

Clinical trial registration	*Provide the trial registration number from ClinicalTrials.gov or an equivalent agency.*
Study protocol	*Note where the full trial protocol can be accessed OR if not available, explain why.*
Data collection	*Describe the settings and locales of data collection, noting the time periods of recruitment and data collection.*
Outcomes	*Describe how you pre-defined primary and secondary outcome measures and how you assessed these measures.*

Dual use research of concern

Policy information about dual use research of concern

Hazards

Could the accidental, deliberate or reckless misuse of agents or technologies generated in the work, or the application of information presented in the manuscript, pose a threat to:

No	Yes	
x		Public health
x		National security
x		Crops and/or livestock
x		Ecosystems
x		Any other significant area

Experiments of concern

Does the work involve any of these experiments of concern:

No	Yes	
☒	☐	Demonstrate how to render a vaccine ineffective
☒	☐	Confer resistance to therapeutically useful antibiotics or antiviral agents
☒	☐	Enhance the virulence of a pathogen or render a nonpathogen virulent
☒	☐	Increase transmissibility of a pathogen
☒	☐	Alter the host range of a pathogen
☒	☐	Enable evasion of diagnostic/detection modalities
☒	☐	Enable the weaponization of a biological agent or toxin
☒	☐	Any other potentially harmful combination of experiments and agents

Plants

Seed stocks	not relevant
Novel plant genotypes	not relevant
Authentication	not relevant

ChIP-seq

Data deposition

☐ Confirm that both raw and final processed data have been deposited in a public database such as GEO.

☐ Confirm that you have deposited or provided access to graph files (e.g. BED files) for the called peaks.

Data access links<br>*May remain private before publication.*	*For "Initial submission" or "Revised version" documents, provide reviewer access links. For your "Final submission" document, provide a link to the deposited data.*
Files in database submission	*Provide a list of all files available in the database submission.*
Genome browser session<br>(e.g. UCSC)	*Provide a link to an anonymized genome browser session for "Initial submission" and "Revised version" documents only, to enable peer review. Write "no longer applicable" for "Final submission" documents.*

Methodology

Replicates	*Describe the experimental replicates, specifying number, type and replicate agreement.*
Sequencing depth	*Describe the sequencing depth for each experiment, providing the total number of reads, uniquely mapped reads, length of reads and whether they were paired- or single-end.*
Antibodies	*Describe the antibodies used for the ChIP-seq experiments; as applicable, provide supplier name, catalog number, clone name, and lot number.*
Peak calling parameters	*Specify the command line program and parameters used for read mapping and peak calling, including the ChIP, control and index files used.*
Data quality	*Describe the methods used to ensure data quality in full detail, including how many peaks are at FDR 5% and above 5-fold enrichment.*
Software	*Describe the software used to collect and analyze the ChIP-seq data. For custom code that has been deposited into a community repository, provide accession details.*

Flow Cytometry

Plots

Confirm that:

- [ ] The axis labels state the marker and fluorochrome used (e.g. CD4-FITC).
- [ ] The axis scales are clearly visible. Include numbers along axes only for bottom left plot of group (a 'group' is an analysis of identical markers).
- [ ] All plots are contour plots with outliers or pseudocolor plots.
- [ ] A numerical value for number of cells or percentage (with statistics) is provided.

Methodology

Sample preparation	*Describe the sample preparation, detailing the biological source of the cells and any tissue processing steps used.*
Instrument	*Identify the instrument used for data collection, specifying make and model number.*
Software	*Describe the software used to collect and analyze the flow cytometry data. For custom code that has been deposited into a community repository, provide accession details.*
Cell population abundance	*Describe the abundance of the relevant cell populations within post-sort fractions, providing details on the purity of the samples and how it was determined.*
Gating strategy	*Describe the gating strategy used for all relevant experiments, specifying the preliminary FSC/SSC gates of the starting cell population, indicating where boundaries between "positive" and "negative" staining cell populations are defined.*

- [ ] Tick this box to confirm that a figure exemplifying the gating strategy is provided in the Supplementary Information.

Magnetic resonance imaging

Experimental design

Design type	*Indicate task or resting state; event-related or block design.*
Design specifications	*Specify the number of blocks, trials or experimental units per session and/or subject, and specify the length of each trial or block (if trials are blocked) and interval between trials.*
Behavioral performance measures	*State number and/or type of variables recorded (e.g. correct button press, response time) and what statistics were used to establish that the subjects were performing the task as expected (e.g. mean, range, and/or standard deviation across subjects).*

Acquisition

Imaging type(s)	*Specify: functional, structural, diffusion, perfusion.*
Field strength	*Specify in Tesla*
Sequence & imaging parameters	*Specify the pulse sequence type (gradient echo, spin echo, etc.), imaging type (EPI, spiral, etc.), field of view, matrix size, slice thickness, orientation and TE/TR/flip angle.*
Area of acquisition	*State whether a whole brain scan was used OR define the area of acquisition, describing how the region was determined.*

Diffusion MRI ☐ Used ☐ Not used

Preprocessing

Preprocessing software	*Provide detail on software version and revision number and on specific parameters (model/functions, brain extraction, segmentation, smoothing kernel size, etc.).*
Normalization	*If data were normalized/standardized, describe the approach(es): specify linear or non-linear and define image types used for transformation OR indicate that data were not normalized and explain rationale for lack of normalization.*
Normalization template	*Describe the template used for normalization/transformation, specifying subject space or group standardized space (e.g. original Talairach, MNI305, ICBM152) OR indicate that the data were not normalized.*
Noise and artifact removal	*Describe your procedure(s) for artifact and structured noise removal, specifying motion parameters, tissue signals and physiological signals (heart rate, respiration).*

Volume censoring | *Define your software and/or method and criteria for volume censoring, and state the extent of such censoring.*

Statistical modeling & inference

Model type and settings | *Specify type (mass univariate, multivariate, RSA, predictive, etc.) and describe essential details of the model at the first and second levels (e.g. fixed, random or mixed effects; drift or auto-correlation).*

Effect(s) tested | *Define precise effect in terms of the task or stimulus conditions instead of psychological concepts and indicate whether ANOVA or factorial designs were used.*

Specify type of analysis: ☐ Whole brain ☐ ROI-based ☐ Both

Statistic type for inference | *Specify voxel-wise or cluster-wise and report all relevant parameters for cluster-wise methods.*

(See Eklund et al. 2016)

Correction | *Describe the type of correction and how it is obtained for multiple comparisons (e.g. FWE, FDR, permutation or Monte Carlo).*

Models & analysis

n/a	Involved in the study
☐	☐ Functional and/or effective connectivity
☐	☐ Graph analysis
☐	☐ Multivariate modeling or predictive analysis

Functional and/or effective connectivity | *Report the measures of dependence used and the model details (e.g. Pearson correlation, partial correlation, mutual information).*

Graph analysis | *Report the dependent variable and connectivity measure, specifying weighted graph or binarized graph, subject- or group-level, and the global and/or node summaries used (e.g. clustering coefficient, efficiency, etc.).*

Multivariate modeling and predictive analysis | *Specify independent variables, features extraction and dimension reduction, model, training and evaluation metrics.*
